# Trend Analysis of Suicide Mortality and Years of Life Lost from 2012 to 2023 in Khuzestan Province, Iran: A Cross-sectional Study

**DOI:** 10.34172/jrhs.11469

**Published:** 2025-10-18

**Authors:** Habibollah Azarbakhsh, Seyed Parsa Dehghani, Elahe Piraie, Mahdiyeh Rashedi, Reza Davasaz-Irani, Fatemeh Rezaei

**Affiliations:** ^1^Social Determinants of Health Research Center, Ahvaz Jundishapur University of Medical Sciences, Ahvaz, Iran; ^2^School of Medicine, Shiraz University of Medical Sciences, Shiraz, Iran; ^3^Social Determinants of Health Research Center, Yasuj University of Medical Sciences, Yasuj, Iran; ^4^Ahvaz Jundishapur University of Medical Sciences, Ahvaz, Iran; ^5^Research Center for Social Determinants of Health, Jahrom University of Medical Sciences, Jahrom, Iran

**Keywords:** Years of life lost, Suicide, Iran, Trend, Mortality rate, Joinpoint regression

## Abstract

**Background:**

The present study was designed to assess the mortality rate and years of life lost due to suicide in Khuzestan province.

**Study Design:**

A cross-sectional study.

**Methods:**

In this study, data on all deaths caused by suicide in Khuzestan province were obtained from the population-based Electronic Death Registration System (EDRS). Crude and age-standardized suicide mortality rates (ASR) were calculated based on gender and year of death over the study period. Subsequently, the number of years of life lost (YLL) was calculated based on age and gender. Joinpoint regression analysis was used to examine the trends in crude mortality rates, age-standardized rates (ASRs), and YLL rates.

**Results:**

During the study years, 1904 suicide deaths occurred in Khuzestan province. Of these deaths, 1157 (60.8%) occurred in men. The highest number of deaths in both genders occurred due to hanging. According to the joinpoint regression, the 12-year trend of YLL rate due to premature mortality was stable. The average annual percentage change (AAPC) was -0.4% (95% CI -4.5 to 7.9, *P*=0.986) for males, and it was 2.4% (95% CI -1.6 to 7.5, *P*=0.222) for females. There were 1 joinpoint and 2 time periods for males (2012-2014 (non-significant decreases) and 2014-2023 (significant increases)), and there were 1 joinpoint and 2 time periods for females (2012-2016 (significant decreases) and 2016-2023 (significant increases)).

**Conclusion:**

The findings revealed a significant increase in age-standardized mortality rate among women and a stable trend among men, and a slight rise in crude mortality rate in men. To address these concerns, it is recommended that targeted region-specific prevention programs be strengthened.

## Background

 According to the definition provided by the World Health Organization (WHO), suicide attempts are actions in which an individual intentionally inflicts harm upon themselves without the interference of others.^1^ Suicide is considered one of the most significant global public health concerns. It ranks as the 13th leading cause of death overall and the third leading cause of death among individuals aged 15–34 years.^2^ Approximately 800­000 suicide-related deaths occur worldwide annually (one death every 40 seconds).^3^ Despite a significant decline in the global suicide rate from 13.8% to 9.8% per 100­000 people between 1990 and 2019, the overall number of suicide deaths has increased by 19­897 cases.^4^ In 2021, there were 746­000 suicide deaths worldwide, 519­000 of which occurred in men.The age-standardized mortality rate has declined over time, from 14.9 deaths (12·8–15·7) per 100 000 population in 1990 to 9.0 (8.3–9.6) per 100 000 in 2021.^5^ This rise imposes significant economic and social costs, resulting in productivity loss and adverse psychological impacts on affected individuals, their social networks, and family members.^6^ Suicide is a complex and multifactorial phenomenon associated with individual, familial, and social factors, including male gender, younger age, and mental disorders such as schizophrenia.^7^ The epidemiological distribution of suicide is influenced by a complex interplay of demographic, cultural, economic, and social factors, as well as time, place, accessibility of means, and so on.^8^ Obtaining accurate data on suicide trends and patterns is challenging, as these data are often underreported due to sociocultural factors.^9^

 Although suicide attempts are generally less prevalent in Islamic countries such as Iran compared to other nations, evidence indicates a rising trend in these regions.^10^ In Iran, statistics show that between 2006 and 2015, a total of 35­279 suicide-related deaths occurred. During this 10-year period, the average total years of life lost (YLLs) due to premature death was 34.52 per 1­000 persons in males, 13.61 per 1000 persons in females, and 23.35 per 1000 persons in both genders. The annual change in the YLL rate was reported to be 3.3%.^11^ The latest reports estimate that the suicide rate in Iran is 6.8 per 100­000, which is lower than the global rate and places the country 58th in the world rankings.^1^ The indicator of YLLs due to premature death is a valuable analytical tool for prioritizing public health concerns. It can be used across different geographic regions by applying time as a measurement unit and comparing years of life lost with the standard life expectancy curve.^12^ Unlike crude mortality rate, the YLL indicator gives more weight to deaths occurring among young adults.^13^

 Khuzestan province is located in southern Iran and has a population of over 4 million, making it the fifth most populous province in Iran. According to the Statistical Center of Iran, Khuzestan is known as the most emigrant-pole in Iran (according to the last census in 2016). Based on available statistics, more than 50% of them have been rural immigrants.^14^ Given that no study has been conducted on mortality rates and years of life lost due to suicide in Khuzestan province, the present study was designed to assess the mortality rate and years of life lost due to suicide in Khuzestan province.

## Materials and Methods

 In this cross-sectional study, data on all deaths caused by suicide in Khuzestan province were obtained from the population-based Electronic Death Registration System (EDRS). Data from health centers and health houses in rural and urban areas, forensic medicine, hospitals, and cemeteries are transmitted to the district health center, where the data is checked with civil registration.^15^ Therefore, the probability of underreporting the number of suicide cases is minimized. The study period spanned from 2012 to 2023. Extracted variables included age at death, year of death, method of suicide, and gender. Inclusion criteria were residency in Khuzestan province and deaths attributed to intentional self-harm. Causes of death were coded based on the 10th revision of the International Classification of Diseases (ICD-10), specifically codes X60–X84, which pertain to suicide. The total estimated population of Khuzestan province was derived using baseline data from health centers and national census data from 2006 and 2016, adjusted based on annual population growth rates. For standardization, the 2013 WHO standard population for low- and middle-income countries was used.^16^

###  Statistical analysis

 First, crude and age-standardized suicide mortality rates (ASR) were calculated based on gender and year of death over the study period. Subsequently, the number of YLLs was computed using standard life tables and life expectancy estimates stratified by age and gender, following a specific formula.^17^ YLL calculations were performed using Microsoft Excel version 2016.

 YLL = N Ce^(ra)^ / (β + r)^2^ [e^-(β + r)(L + a)^ [-(β + r) (L + a)-1] – e^-(β + r)a^ [–(β + r) a-1]]

 N represents the number of deaths in each age group and gender. L denotes the standard life expectancy corresponding to that age and gender, and r is the discount rate, set at 0.03. β is the age-weighting parameter, conventionally fixed at 0.04. C is a constant adjustment factor with a value of 0.1658, a indicates the age at death, and e is the base of the natural logarithm, fixed at 2.71.

 βdetermines the most valuable ages, and by changing it, the age that yields the most value can be changed. C is an adjustment constant and is chosen so that the age weights do not change the total number of years of life lost. To estimate net present value and years of life lost, the Global Burden of Disease study assumes a 3% discount rate.^18^

 Joinpoint regression analysis was used to examine the trends in crude mortality rates, ASRs, and YLL rates. This method identifies time segments with distinct trends and estimates annual percentage changes (APCs) for each segment based on the slope of the fitted line. Then, the average annual percentage change (AAPC) was calculated to summarize the overall trend.^19^ This method is a statistical technique used in time series modeling and is widely used in epidemiological studies to model the time trend of incidence and mortality data. It identifies the number (if any) and location of changes in the trend (joinpoints).^20^ This joinpoint analysis of the trend was carried out by Joinpoint Regression Program version 5.3.0.0.

## Results

###  Mortality rate due to suicide

**Figure 1:**
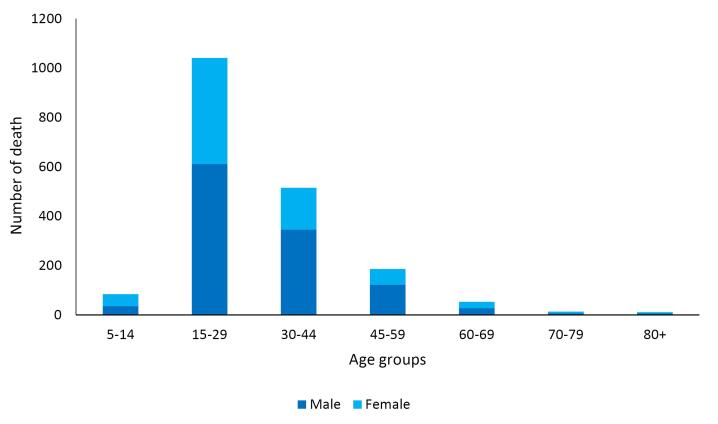
Number of deaths due to suicide by gender and age groups during 2012-2023

**Figure 2:**
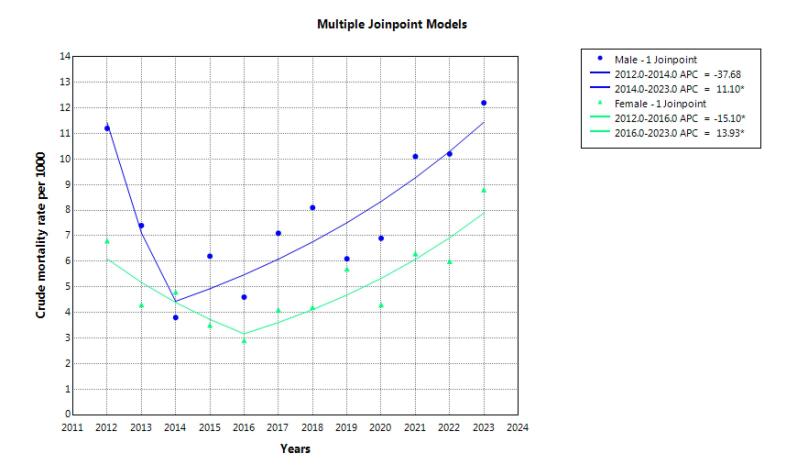
The trend of crude mortality rate due to suicide in males and females during 2012-2023

**Figure 3:**
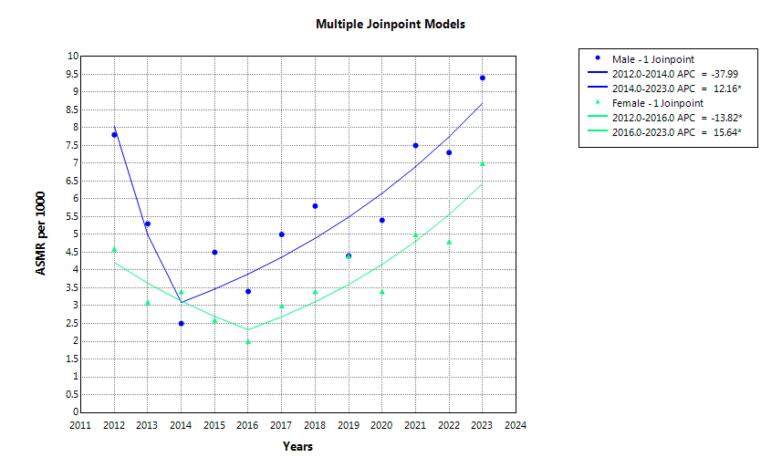
The trend of age-standardized mortality rate due to suicide in males and females during 2012-2023

 During the study years, 1904 suicide deaths occurred in Khuzestan province. A total of 1157 deaths (60.8%) occurred in men. The highest number of deaths in both genders was observed in the 15-29 age group (Figure 1). The highest number of deaths in both genders occurred due to hanging. The crude mortality rate in men increased from 11.2 in 2012 to 12.2 in 2023, but it was not statistically significant. AAPC was 0.1% (95% CI -4.5 to 9.0, *P* = 0.849). The crude mortality rate in women also had a constant trend, 8.6 in 2012 and 8.8 in 2023, and AAPC was 2.4% (95% CI -1.2 to 6.9, *P* = 0.190) (Table 1, Figure 2). The trend in age-standardized mortality rates has been stable in men but increasing in women. AAPC was 0.7% (95% CI -3.3 to 8.8, *P* = 0.668) and 3.9% (95% CI 0.9 to 7.2, *P* = 0.006) for males and females, respectively (Table 1, Figure 3).

**Table 1:** The Trend of crude and age-standardized mortality rates (per 100­000 population) and years of life lost due to suicide by gender and year in Khuzestan province during 2012-2023

**Year**	**Death (n)**		**Crude mortality rate**		**ASMR (95%CI)**		**Years of life lost**			
							**Death (n)**		**(per 1000)**	
	**Male**	**Female**	**Male**	**Female**	**Male**	**Female**	**Male**	**Female**	**Male**	**Female**
2012	134	80	11.2	6.8	7.8 (6.3, 9.3)	4.6 (3.5, 5.8)	3426	2079	2.9	1.8
2013	88	51	7.4	4.3	5.3 (4.1, 6.5)	3.1 (2.2, 4.1)	2224	1347	1.9	1.1
2014	45	55	3.8	4.8	2.5 (1.6, 3.3)	3.4 (2.4, 4.3)	1156	1435	1.0	1.2
2015	74	42	6.2	3.5	4.5 (3.4, 5.6)	2.6 (1.7, 3.4)	1875	1097	1.6	0.9
2016	56	34	4.6	2.9	3.4 (2.4, 4.3)	2.0 (1.2, 2.7)	1389	933	1.2	0.8
2017	86	49	7.1	4.1	5.0 (3.9, 6.2)	3.0 (2.1, 3.9)	2185	1291	1.8	1.1
2018	99	50	8.1	4.2	5.8 (4.6, 7.0)	3.4 (2.5, 4.3)	2503	1283	2.0	1.1
2019	76	69	6.1	5.7	4.4 (3.3, 5.5)	4.4 (3.2, 5.4)	1929	1788	1.6	1.5
2020	86	53	6.9	4.3	5.4 (4.3, 6.6)	3.4 (2.5, 4.3)	2106	1369	1.7	1.1
2021	127	78	10.1	6.3	7.5 (6.1, 8.9)	5.0 (3.9, 6.1)	3122	2081	2.5	1.7
2022	130	75	10.2	6.0	7.3 (5.9, 8.6)	4.8 (3.7, 5.8)	3218	1895	2.5	1.5
2023	156	111	12.2	8.8	9.4 (8.0, 10.9)	7.0 (5.7, 8.2)	3940	2867	3.1	2.3
Total	1157	747	7.9	5.2	5.7 (5.3, 6.1)	3.9 (3.6, 4.2)	29073	19465	2.0	1.3
*P*-value	-	-	0.849	0.190	0.668	0.006	-	-	0.986	0.222

###  YLL due to suicide

**Figure 4:**
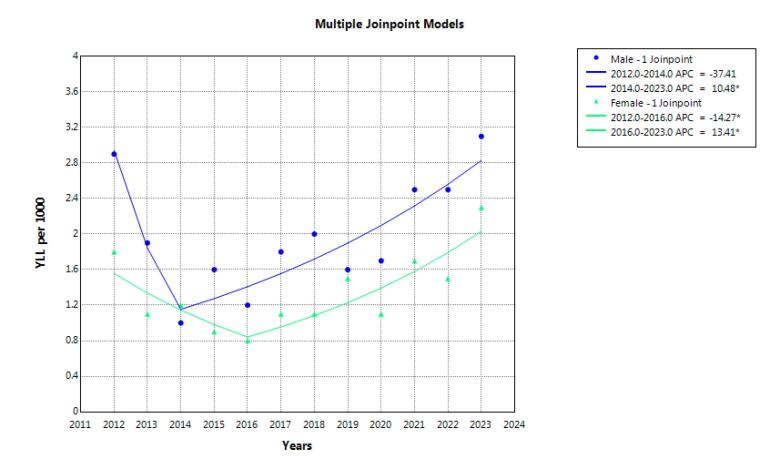
The trend of years of life lost due to suicide in males and females during 2012-2023

 The total number of years of life lost due to suicide during the study years was 48­538 years, and it was 29073 (59.9%) years in men. The highest number of years of life lost in both genders was attributed to hanging. According to the joinpoint regression, the 12-year trend of YLL rate due to premature mortality was stable. AAPC was -0.4% (95% CI -4.5 to 7.9, *P* = 0.986) for males, and it was 2.4% (95% CI -1.6 to 7.5, *P* = 0.222) for females. There were 1 joinpoint and 2 time periods for males. In one period from 2012 to 2014 (the first period), non-significant decreases in YLL rate were seen, and the APC was -37.4% (95% CI -50.6 to 2.3, *P* = 0.068). In the other period from 2014 to 2023 (the second period), significant increases in YLL rate were seen, and the APC was 10.5% (95% CI 3.3 to 41.8, *P* = 0.024). There were 1 joinpoint and 2 time periods for females. In one period from 2012 to 2016 (the first period), significant decreases in YLL rate were seen, and the APC was -14.3% (95% CI -36.9 to -0.0, *P* = 0.050). In the other period from 2016 to 2023 (the second period), significant increases in YLL rate were seen, and the APC was 13.4% (95% CI 6.0 to 43.7, *P* = 0.006) (Figure 4).

## Discussion

 Findings over the 12-year period revealed a slight increase in the crude mortality rate among men, though this change was not statistically significant. Among women, the crude mortality rate remained relatively stable. However, the age-standardized mortality rate (ASMR) showed a significant increase among women, while remaining stable among men. Although the overall YLL trend remained relatively steady, Joinpoint regression analysis revealed two distinct phases: an initial decline followed by a significant increase in YLL rates, beginning in 2014 for men and 2016 for women.

 The results showed that over a 12-year period, the crude mortality rate increased from 11.2 to 12.2 per 100­000 in men, but it remained almost constant in women. During the same period, age-standardized mortality rates showed a significant increase in women but no significant change in men. The global ASMR due to suicide was estimated to be 9 per 100­000 in 2019, with a notably higher rate among men (12.6) compared to women (5.4).^21^ Reports indicate that the suicide rate in Iran is 6.8 per 100­000, ranking 58th globally.^22^ Notably, the global ASMR has been decreasing.^23^ Zabihi Afroozi et al found that although suicide attempts were more frequent among women, completed suicides were more common among men, indicating a higher mortality rate due to suicide among males.^24^ Shakibkhah et al also reported increasing trends in both crude and standardized suicide mortality among men, with stable rates among women.^25,26^. In a systematic review and meta-analysis, Rouzrokh et al showed that the overall suicide mortality rate was higher among men in Iran, especially non-western men. However, Iranian women in western provinces, especially through self-immolation, showed disproportionately higher suicide rates.^26^ Similar trends were reported by Park et al in South Korea, indicating increased suicide rates among women over the past decade, attributed in part to rising socio-economic pressures.^27^ Suicide is a multifactorial phenomenon influenced by cultural, social, and economic factors.^26,28^ The observed gender differences, particularly the significant increase in female suicide mortality, may be attributed to unique sociocultural structures, lack of social support, domestic violence, the use of violent methods such as self-immolation, entrenched gender biases, restricted freedoms, unemployment, and economic hardship. It is noteworthy that women may encounter unique pressures and challenges that are different from those of men. Women face greater challenges, such as gender-based violence and discrimination in career advancement. In addition, societal and familial expectations to balance career and family responsibilities are among the main challenges women face.^29^ Therefore, women’s needs should be considered and intervention and educational programs should be implemented to reduce and prevent suicide if necessary. Regional variations may also stem from differences in geography, customs, and lifestyle across Iranian provinces. It is plausible that certain regions harbor specific local risk factors. These findings underscore the need for regionally tailored prevention policies that are sensitive to local cultural and social contexts.

 This study confirmed that the highest burden of suicide mortality and YLL occurred among individuals aged 15–29 years, with hanging as the most frequent method of suicide in both genders. These findings align with previous studies conducted in Iran.^25^ For instance, Shafiee-Kandjani et al reported an overall suicide mortality rate of 8.14 per 100­000, with higher rates observed among men (9.1) compared to women (5.0), and hanging as the most common method.^30^ Similarly, in a multicenter study, Fathi et al highlighted the predominance of youth in suicide-related deaths and confirmed that hanging was the most widespread method across regions.^31^ There is growing concern about rising suicide rates among Iranian youth, particularly young physicians, which may reflect increasing socio-economic stressors.^32^ The leading suicide methods vary globally due to differential access to lethal means and cultural-behavioral differences across countries and even within subnational regions.^33^ Suicide behavior is inherently multifactorial and cannot be attributed to a single determinant. Personality traits may also play a significant role.^34^. These findings highlight the urgent need for targeted interventions and enhanced mental health infrastructure, particularly for vulnerable youth populations. A better understanding of suicide patterns by age, gender, method, and socioeconomic status can support more effective preventive policies and reduce the public health burden.

 Trend analysis of YLL over time is critical for identifying major shifts in population health and guiding policymakers in designing targeted interventions. Joinpoint regression serves as a valuable tool for detecting trend changes and identifying periods of rising or declining YLL rates, thereby enabling timely and appropriate responses.^20,35^ Our analysis revealed two distinct Joinpoint phases: an initial decline in YLL for both genders, followed by a significant increase from 2014 in men and 2016 in women. These shifts may reflect underlying social, economic, or healthcare-related changes. The early reduction in YLL may be linked to improved healthcare access, increased public awareness, or successful early prevention programs.^36^ Conversely, the subsequent increase could be attributed to rising psychological and social pressures in Iran and other middle-income countries.^37,38^ Economic and social stressors likely contributed to higher rates of premature mortality from suicide and psychosomatic diseases, thereby raising overall YLL.^23,37^ In addition, demographic transitions, such as the rising youth or elderly population, can alter disease burden and influence YLL. A decline in the effectiveness of existing prevention programs may also contribute to this upward trend, suggesting a need for review and recalibration.

 Iran’s national suicide prevention program became a part of primary health care in 2010. The goal of this program is to improve suicide prevention efforts across the country.^39^ However, a national commitment is necessary to decrease the prevalence of suicide through the promotion of good mental health, the reduction of poverty and inequality, the prevention of alcohol and drug use, and the promotion of social justice and human rights.^40^

 The present study has several strengths. First, its longitudinal design over a 12-year period enabled precise identification of temporal changes and turning points in trends. Second, the use of advanced Joinpoint regression analysis offered deeper insights into significant trend shifts, including periods of increase or decline. Third, the application of multidimensional indicators, including crude and age-standardized mortality rates and YLL, provided a comprehensive evaluation of the suicide burden. Nevertheless, several limitations exist. Suicide may be underreported due to social stigma, religious beliefs, and legal implications. The lack of contextual data, such as psychiatric history, socioeconomic status, and access to mental health services, limits our ability to fully understand associated risk factors. Furthermore, given that the study was conducted solely in Khuzestan province, generalizability of the findings to other regions of Iran may be limited due to cultural and economic differences. Finally, reliance on official death registration data may result in the omission of unregistered suicide cases.

**Highlights**

- During the study years, 1904 suicide deaths occurred in Khuzestan province.
- The total YLL due to suicide was 29­073 in males and 19­465 in females.
- According to the joinpoint regression analysis, the 12-year trend of YLL rate due to premature mortality was stable.

## Conclusion

 The findings revealed a significant increase in age-standardized mortality among women, a relatively stable trend among men, and a slight rise in crude mortality in men. The highest burden of suicide mortality and YLL was observed in the 15–29 age group, with hanging identified as the most common suicide method in both genders. Joinpoint regression analysis identified two distinct phases, including an initial decline in YLL followed by a statistically significant increase beginning in 2014 for men and 2016 for women, likely influenced by social, economic, or healthcare dynamics. Since suicide is a multifactorial phenomenon, social, economic, and cultural interventions are needed to prevent and reduce suicide.

## Acknowledgments

 We would like to acknowledge the Health Vice-chancellor, Ahvaz Jundishapur University of Medical Sciences for providing the resources.

## Competing Interests

 The authors declare that they have no conflict of interests related to the publication of this study.

## Ethical Approval

 The protocol of this study was reviewed and approved by the Ethics Committee of Ahvaz Jundishapur University of Medical Sciences (IR.AJUMS.REC.1402.654). Given the nature of the data and the lack of personal information, there was no need for informed consent. All procedures were conducted in accordance with the ethical code of the university and research standards.

## Funding

 None.
